# Annotations

**Published:** 1895-12-14

**Authors:** 


### Annotations.

### A Sanitary Record for Hull.

For some reason an impression has generally prevailed that Hull is an unhealthy town. It lies low on the banks of the Humber; and its drainage system must suffer from a deficient fall. Nevertheless the zymotic death-rate of Hull is remarkably low, and the small number of deaths from diphtheria during 1894 and for a good many previous years suggests an immunity from this particular disease which is almost phenomenal. Dr. Wright Mason, the medical officer of health for the borough, adopts several "graphic" methods for depicting the comparative rates of mortality from the principal causes of diseases. In one table he shows by solid columns of varying heights the rates for the twenty principal causes. The shortest of all these columns is the one which represents diphtheria. It will scarcely be believed, nevertheless it is the fact, that in Hull last year measles killed four times as many people as diphtheria, whooping cough three and a-half times as many, and even diarrhœa more three times as many. This is of special importance, in connection with the steadily increasing prevalence of diphtheria, in London. Why should it advance among us with ever-growing strides, whilst in Hull it has remained more or less stationary, or even diminished, during so considerable a period as twenty years? This is a question which the sanitary authorities of the metropolis will do well to put to themselves, and to which they should seek a decisive answer. It cannot be that Hull has no Board schools. She must have as many in proportion to population as London. On the general question of his annual report, and on the beauty and efficiency of the "graphic" methods he has employed, we desire to offer our sincere congratulations to Dr. Wright Mason.

### How to Get into a Fever Hospital.

The unfortunate misunderstanding which arose last week between the authorities at the Poplar Hospital and the officials of the Metropolitan Asylums Board makes it clear that medical men, who in such matters have to be the teachers and instructors of the public, do not quite recognise what is the position of affairs at Norfolk House in times of pressure. So long as there are plenty of beds the old rule holds good, and a mere telegram will fetch an ambulance, and if on arrival the nurse finds a certificate stating the nature of the case and certifying that the patient is fit for removal, removal will at once take place. But in times of pressure, when there are not enough beds to go round, it is necessary to make some selection, and a moment's consideration is enough to make it obvious that for this purpose a certain amount of information must be given besides mere name, age, and sex, important as these are. Again it is clear that, with people clamouring on all sides for admission, it is impossible to reserve beds, nor is it possible to admit cases in the order of their application. If that were done we fear the Board would be so far in arrear as to be dealing now with cases that occurred weeks and weeks ago, and would be entirely throwing away all its possibilities of usefulness. All that the Board at present finds itself able to do is each morning to select what appear to be the most urgent cases. For this purpose it is absolutely necessary to have both reliable and recent information, hence the rule that daily application must be made. We would therefore urge upon medical men the absolute importance, in times when there is a strain upon the hospitals, not to be content with a mere application, but to inform the officials at Norfolk House of all those details which denote urgency, and in case of failure to obtain admission to renew the application each morning so long as admission may seem desirable. It is only thus that it is possible for the officials to use the beds to the best advantage. This, then, is the way to get into a fever hospital in London.

### The Clinics of the Future.

"Le Scalpel" is, as its name implies, a medical paper, but it is not above printing a *feuilleton*, and in a recent number it devotes this lighter page to a fanciful description of the clinical teaching of the future. The suggestion is, that for teaching purposes there are many phenomena which can be more thoroughly explained, and, in fact, be more easily understood, by means of the sort of analysis rendered possible by means of such instruments as the kinetograph, phonograph, &c., than by observation of the actual patient. Taking, for a moment, the kinetograph as an example, it is clear that there are a very large number of morbid states which, although common enough, occur at inconvenient seasons, and are extremely difficult to demonstrate to a class. The whole range of hysteria, with its tremblings, its convulsions, its passionate attitudes and catalepsies, is practically a sealed book to the average student who has not held a resident appointment. How different all this would be with the kinetograph! Good slides once taken would be at the disposal of the world for teaching purposes. An epileptic fit and an attack of grand hysteria could be demonstrated in immediate succession and be readily compared. By its aid also it would be unnecessary to make the hemiplegic drag his weary limbs, or the spastic paraplegic hop along, or the case of athetosis go through his performances; all would be done by the machine, and instead of being seen only as chance might determine in the wards, they would all take their proper places in the lectures on the subjects in question. The phonograph also is to be made full use of. No longer will the weary patient be made to cough, cough, cough, so that the stupid student may be taught to recognise the morbid sounds within his chest. Râles and voice sounds, dulness and resonance, and all the various heart sounds will be taught upon the phonograph, as also will the epileptic cry, the clanging cough of croup, and the drawling or staccato speech of different forms of paralysis. We need not follow *Le Scalpel* in its ingenious endeavour to depict the clinical lecture of the future. In its way, it is excellent fooling, but it covers a truth, and there can be no possible doubt that by the adoption of some such methods as are here suggested medical lectures could be made far more interesting and useful than they too often are at present.

